# A Comparative Study of Rutin and Rutin Glycoside: Antioxidant Activity, Anti-Inflammatory Effect, Effect on Platelet Aggregation and Blood Coagulation

**DOI:** 10.3390/antiox10111696

**Published:** 2021-10-27

**Authors:** Sung-Sook Choi, Hye-Ryung Park, Kyung-Ae Lee

**Affiliations:** 1 Department of Food and Nutrition, Duksung Women’s University, Seoul 01370, Korea; choiss@duksung.ac.kr; 2 Graduate School of Biotechnology, College of Life Science, Kyung Hee University, Yongin 17104, Korea; hrpark8512@khu.ac.kr; 3 Department of Food and Nutrition, Anyang University, Anyang 14028, Korea

**Keywords:** rutin, rutin glycoside, transglycosylation, solubility, antioxidant, anti-inflammatory, platelet aggregation, blood coagulation, in vitro, in vivo

## Abstract

The effects of rutin and rutin glycoside with different solubility were compared on antioxidant activity and anti-inflammatory effects in vitro and the effects on platelet aggregation and blood coagulation in vitro and in vivo. Rutin glycoside (consisting of rutin mono-glucoside and rutin di-glucoside) was prepared via enzymatic transglycosylation from rutin. Rutin glycoside showed a higher effect than rutin on radical scavenging activity in antioxidant assays. Rutin showed a higher toxicity than rutin glycoside in murine macrophage RAW264.7 cells. They had similar effects on the levels of nitric oxide (NO), prostaglandin E (PGE) 2 and pro-inflammatory cytokines (such as tumor necrosis factor (TNF)-α, and interleukin (IL)-6) in the cells. Both rutin and rutin glycosides similarly reduced the rate of platelet aggregation compared to controls in vitro. They also similarly delayed prothrombin time (PT) and activated partial thromboplastin time (APTT) in an in vitro blood coagulation test. The effect of repeated administration of rutin and rutin glycoside was evaluated in vivo using SD rats. The platelet aggregation rate of rutin and the rutin glycoside administered group was significantly decreased compared to that of the control group. On the other hand, PT and APTT of rutin and rutin glycoside group were not significantly delayed in vivo blood coagulation test. In conclusion, rutin and rutin glycoside showed differences in antioxidant activities in vitro, while they were similar in the reduction of NO, PGE2, TNF-α and IL-6 in vitro. Rutin and rutin glycoside also showed similar platelet aggregation rates, and blood coagulation both in vitro and in vivo condition. Comparing in vitro and in vivo, rutin and rutin glycoside were effective on platelet aggregation both in vitro and in vivo, but only in vitro on blood coagulation.

## 1. Introduction

Rutin (3,3′,4′,5,7-pentahydroxyflavone-3-rhamnoglucoside) is a flavonol, found abundantly in plants such as the flowers of pagoda tree, buckwheat seeds, tea and berries. Chemically, it is a glycoside composed of flavonol aglycone quercetin along with the disaccharide rhamnosyl glucose. Rutin is well known as a potent natural antioxidant effective in lowering oxidative stress [1,2]. It has demonstrated many pharmacological activities of rutin, including anti-inflammatory, antimicrobial, anticarcinogenic, neuroprotective, antithrombotic and anti-viral activities [3,4]. However, the low water solubility of rutin limits to its expansion into food and pharmaceutical applications. Various studies have been conducted to increase the water solubility and efficacy of rutin. Rutin encapsulated with β-cyclodextrin was studied for its solubility and biological activity by some research groups [5,6]. Enzymatically polymerized rutin was investigated for its antioxidant activity and wound healing potential [7]. Chemical and enzymatic modifications of rutin have also been studies. Pedriali et al. chemically synthesized water-soluble rutin derivatives and reported their antiradical effect [8], and Lee et al. synthesized rutin derivatives via enzymatic glycosylation using *Penicillium decumbens* naringinase and studied their increased solubility and activity [9].

**Figure 1:**
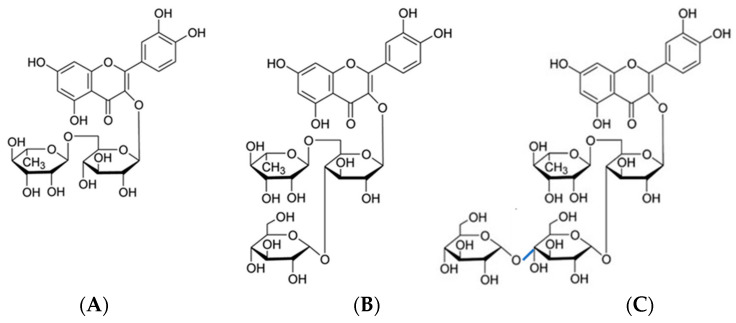
Chemical structures of rutin and its glycosylated compounds. (**A**) Rutin, (**B**) rutin mono-glucoside and (**C**) rutin di-glucoside.

Glycosylation is one of the promising technologies available to improve the solubility and stability of natural compounds [10]. An increase in antibacterial activity and α-glucosidase inhibition were reported for hesperidin glycoside [11]. The *O*-glycosylation of resveratrol was reported to increase both its solubility in water and bioavailability while preventing oxidation [12]. The glycosylation of rutin has been extensively studied and is considered an efficient and available method to increase solubility [13,14,15]; however, information on the changes in its properties and efficacy is not fully understood. This study was conducted to investigate whether rutin and rutin glycoside with different solubilities may have different effects on antioxidant, anti-inflammation and antithrombotic activities (such as platelet aggregation and blood coagulation). At the same time, the effects of rutin and rutin glycoside between in vitro and in vivo conditions were compared on platelet aggregation and blood coagulation. The chemical structures of rutin and its glycosides (rutin mono-glucoside and rutin di-glucoside) are shown in Figure 1.

## 2. Materials and Methods

### 2.1. Materials and Chemicals

Purified rutin powder (>95%) isolated from *Sophora japonica* was purchased from Natural Biotech. Co. (Seoul, Korea). Commercial enzymes, cyclodextrin glucanotransferase (CGTase, Toruzyme), glucoamylase (AMG) and β-amylase (Secura) were products of Novozymes (Gladsaxe, Denmark). Dextrin with dextrose equivalent (DE) value 8–15 was a product of Sigma-Aldrich Co. (St. Louis, MO, USA) and an adsorption resin (Amberlite XAD-7) was a product of Dow Chemical (Midland, MI, USA).

Reagents for antioxidant assay, 1,1-Diphenyl-2-picrylhydrazyl (DPPH) and 2,2′-azino-bis-(3-ethylbenzothiazoline-6-sulfonic acid) (ABTS) were purchased from Sigma-Aldrich Co. (St. Louis, MO, USA). For a cell based assay, penicillin/streptomycin solution (each 10,000 U/mL) was purchased from Thermo Fisher Scientific (Waltham, MA, USA), fetal bovine serum (FBS) and Dulbecco’s modified eagle’s medium (DMEM) were purchased from Hyclone (Logan, UT, USA). Dimethyl sulfoxide (DMSO), lipopolysaccharide (LPS) and 3-(4,5-dimethylthiazol-2-yl)-2,5-diphenyltetrazolium bromide (MTT) were products of Sigma Chemical Co. (St. Louis, MO, USA). A nitric oxide (NO) detection kit was purchased from iNtRON Biotechnology (Seongnam, Korea) and an enzyme-linked immunosorbent assay (ELISA) kit of prostaglandin E (PGE) 2 was purchased from Enzo Biochem. (New York, NY, USA) and kits of tumor necrosis factor (TNF)-α and interleukin (IL)-6 were purchased from Thermo Fisher Scientific. Aspirin (acetylsalicylic acid) was purchased from Sigma-Aldrich Co. (St. Louis, MO, USA) and heparin (injection grade, 5000 IU/mL) was a product of JW Pharmaceutical corp. (Seoul, Korea).

### 2.2. Preparation of Rutin Glycoside

For the preparation of rutin glycoside, rutin powder was dissolved at a concentration of 1% (*w*/*v*) in 50% ethanol solution at 60 °C. Dextrin was added to the solution in a ratio of 4:1 to rutin, and the solution was evaporated to reduce ethanol to 10% or less. At the same time, CGTase was added to the solution at a final concentration of 5% (*w*/*v*) and reacted at 50 °C, pH 6.0, for 48 h. After the reaction, a glucoamylase or β-amylase was added to the solution at a concentration of 1% and reacted at 40 °C, pH 6.0, for 3 h to produce rutin mono-glucoside or rutin mono-/di-glucoside, respectively. The reactant was filtered using a membrane filter (0.45 um) and the filtrate was loaded on a column filled with an adsorption resin, Amberlite XAD-7 (Sigma-Aldrich Co., St. Louis, MO, USA). After loading, it was washed with deionized water and eluted with 60% ethanol solution. The eluent was evaporated and concentrated at 50 °C, to reach the final solid concentration of 50%, and five volumes of ethanol were added and cooled to 5 °C or less for more than 24 h to induce precipitation. The final precipitate was collected and dried.

The conversion rate of rutin glycoside from rutin was calculated as a percentage of the amount of rutin reacted after the reaction from the amount of rutin initially used.

$$\mathrm{Conversion}\;\mathrm{rate}\;\left(\%\right)=\frac{\mathrm{Amount}\;\mathrm{of}\;\mathrm{rutin}\;\mathrm{before}\;\mathrm{reaction}-\mathrm{Amount}\;\mathrm{of}\;\mathrm{rutin}\;\mathrm{after}\;\mathrm{reaction}}{\mathrm{Amount}\;\mathrm{of}\;\mathrm{rutin}\;\mathrm{before}\;\mathrm{reaction}}\;\times \;100$$

### 2.3. Analysis of Rutin and Rutin Glycoside

#### 2.3.1. Determination of Rutin and Rutin Glycoside

The composition and content of rutin and rutin glycoside were analyzed via HPLC (Chromaster 5110, Hitachi, Japan). An amide column (TSK Gel Amide-80, 5 μm, 4.6 × 250 mm, Tosoh corp., Tokyo, Japan) was used for the analysis, and the mobile phases were acetonitrile and water binary eluents under gradient conditions: 0 min (80%:20%), 15 min (65%:35%), 30 min (60%:40%), 37 min (80%:20%) and 50 min (80%:20%). The flow rate was 0.8 mL/min, the column temperature was 25 °C, the injection volume was 10 μL and the sample was detected at 214 nm using a UV/Vis spectrophotometer (S22, Biochrom, Cambridge, UK).

The rutin content of the rutin glycoside sample was also measured by the KFDA method [16]. Briefly, the sample was solubilized in deionized water to a concentration of 1% (*w*/*v*), and 1 mL aliquot was diluted with 0.085% phosphoric acid solution to 100 mL. Rutin standard was solubilized in methanol to a final concentration of 1% (*w*/*v*), and 1 mL aliquot was diluted with 0.085% phosphoric acid solution to 100 mL. Phosphoric acid 0.085% solution was used as a control solution and the absorbance of the sample and standard solution was measured at a wavelength of 351 nm using a UV/Vis spectrophotometer (S22, Biochrom, Cambridge, UK). The rutin content of rutin glycoside was calculated as follows:

$$\mathrm{Rutin}\;\mathrm{content}\;\left(\%\right)=\frac{\mathrm{Absorbance}\;\mathrm{of}\;\mathrm{sample}\;\mathrm{solution}\;\times \;\mathrm{Standard}\;\mathrm{amount}\;(\mathrm{mg})}{\mathrm{Absorbance}\;\mathrm{of}\;\mathrm{standard}\;\mathrm{solution}\;\times \;\mathrm{Sample}\;\mathrm{amount}\;(\mathrm{mg})}$$

#### 2.3.2. Identification of Rutin and Rutin Glycoside

Rutin mono-glucoside and di-glucoside in rutin glycoside formed in the reaction were identified by measuring molecular weight through LC-MS-MS analysis. The chromatographic separation using an HPLC system (LC-20AD, Shimadzu, Kyoto, Japan) was performed based on the rutin glycoside analysis method. The HPLC system was directly interfaced with a Q-TOF Premierometer (Waters, Milford, MA, USA) equipped with an electrospray ion source operated in the negative mode. The confirmation ion transitions for quantification were m/z 771 (precursor ion) → 301 (product ion) for rutin monoglycoside and 933 (precursor ion) → 300 (product ion) for rutin di-glycoside.

### 2.4. Determination of Partition Coefficient

The solubility properties of rutin and rutin glycosides were investigated by measuring partition coefficients [17,18]. Octanol–water partition coefficients were determined after mixing the samples (500 μM) in water with the same volume of n-octanol and maintaining the mixture in the dark at room temperature for 20 h to reach partition equilibrium. Flavonoid concentrations in the aqueous phase were determined via HPLC and partition coefficients (log P) were calculated as log ratio of the concentration in the octanol phase to the concentration in the aqueous phase:

$$\mathrm{Partition}\;\mathrm{coefficient}\;\mathrm{Log}\;\left(P\right)=\frac{\mathrm{Log}\;\left[C\right]\mathrm{octanol}\;}{\mathrm{Log}\;\left[C\right]\mathrm{water}}$$

where C_octanol_ is the concentration of the compound in the n-octanol phase and C_water_ is the concentration of the compound in the aqueous phase.

### 2.5. Determination of Antioxidant Activity

#### 2.5.1. DPPH Assay

Determination of radical scavenging activity using DPPH was performed according to the method of Ratha et al. [19] and Blois [20] with slight modification. DPPH was solubilized with absolute ethanol to final concentration of 0.4 mM, and each 0.2 mL of diluted sample was added to 3.8 mL of the DPPH solution. After reaction for 20 min, optical density was measured at 525 nm using a microplate reader (Epoch2, Bio Tek, Winooski, VT, USA). The scavenging activity of DPPH radical was calculated as a percentage of the difference between the sample and control.

#### 2.5.2. ABTS Assay

Radical scavenging activity using ABTS was determined based on the method of Re et al. [21]. ABTS radical solution (7 mM) was mixed with potassium persulfate solution (140 mM) and reacted for 12 h. The solution was diluted with absolute ethanol until the absorbance of 734 nm reached 0.700 ± 0.02 and used as ABTS+ reagent. An aliquot of 0.2 mL of sample solution was added to 5 mL of ABTS+ reagent and mixed for 6 min at room temperature. The absorbance of the reaction solution was measured at 734 nm in a 96-well plate. The scavenging activity of the ABTS radical was expressed as a percentage of difference of sample compared to control.

### 2.6. Determination of Effects on NO, PGE2 and Pro-Inflammatory Cytokine

#### 2.6.1. Cell Culture and Cytotoxicity Assay

Murine macrophage cell line (RAW 264.7) was provided from Korea Cell Line Bank (Seoul, Korea). Cells were maintained in DMEM supplemented with 10% FBS and a mixture of penicillin and streptomycin (100 μg/mL each). An MTT assay [22] was used to assess the cytotoxicity of rutin and rutin glycoside. Cells were cultured in 96-wells at a density of 5 × 10^4^ cells/well and incubated for 24 h in an incubator with 5% CO_2_ atmosphere. Each sample of rutin and rutin glycoside at different concentrations was placed in the well and maintained for 1 h, then LPS (1 μg/mL) was added and incubated for 24 h. After the incubation, the medium was removed and 100 μL of MTT solution was added to the well and maintained at 37 °C for 2 h. DMSO was further added to the reaction mixture and the absorbance was measured at 540 nm in a microplate reader. Cell viability was expressed as a percentage of difference of absorbance at 540 nm of a test sample compared to the control.

#### 2.6.2. Measurement of NO and PGE2 Levels

NO and PGE2 levels were determined as anti-inflammatory intermediates in RAW 264.7 cell line. Cells were cultured in 96-wells at a density of 5 × 10^4^ cells/well and treated with various concentrations of rutin and rutin glycoside for 1 h at 37 °C. Then, LPS was added to each well at final concentration of 1 μg/mL and incubated for 24 h, and 100 μL aliquot of each medium was mixed with 100 μL of Griess reagent in a new well. After reaction for 15 min, the absorbance value was read at a wavelength of 540 nm in a microplate reader. Nitrite concentration in the reactant was obtained from a standard curve of sodium nitrite.

Cell culture conditions for PGE2 assay were the same as those used for the NO assay. PGE2 level in the media was measured using an ELISA kit according to the manufacturer’s instruction. The absorbance value was read at a wavelength of 405 nm in a microplate reader.

#### 2.6.3. Measurement of Pro-Inflammatory Cytokine Levels

The anti-inflammatory activity of rutin and rutin glycosides was also evaluated by measuring the levels of TNF-α and IL-6 in RAW 264.7 cells. Cell culture conditions for TNF-α and IL-6 assay were the same as those used for the NO or PGE2 assay. After incubation for 24 h with samples and LPS, the concentrations of TNF-α and IL-6 in the media were measured respectively, using commercial detection kits according to the manufacturer’s instruction. The absorbance value was read at a wavelength of 450 nm in a microplate reader.

### 2.7. Platelet Aggregation and Blood Coagulation In Vitro and In Vivo

#### 2.7.1. Condition for In Vitro Test

In vitro platelet aggregation and blood coagulation tests were approved by the Institutional Animal Care and Use Committee of Osong Medical Innovation Foundation (No. KBIO-IACUC-2020-208).

Male Sprague–Dawley Crl:CD rats were provided by Orient Bio (Seongnam, Korea). Temperature and relative humidity were maintained at 22 ± 2 °C and 50 ± 10%, respectively, and commercial rodent pellet feed and drinking water were supplied before the experiment. The rats weighing 289.3–326.0 g were grouped into four groups with four rats in each group; the normal control group (distilled water), the Rutin group, the Rutin glycoside group and the positive control group.

Rutin 0.061 g was dissolved in DMSO to a total amount of 1 mL as 100 mM solution, and rutin glycoside 0.085 g was dissolved in water to a total amount of 1 mL as 100 mM solution. Positive control for platelet aggregation, aspirin 2.5 mg was dissolved in DMSO to a total of 100 μL as a 25 mg/mL solution. Positive control for blood coagulation, heparin 3 µL was diluted with 97 µL of water to prepare a 150 IU/mL solution.

#### 2.7.2. Condition for In Vivo Test

In vivo platelet aggregation and blood coagulation tests were approved by Institutional Animal Care and Use Committee of Osong Medical Innovation Foundation (KBIO-IACUC-2020-173).

The same rat species and condition as the in vitro experiment was used for in vivo experiment. The rats weighing 230.8–258.0 g were grouped into six groups with eight rats in each group; the normal control group (distilled water), the 2 Rutin group, the 2 Rutin glycoside group and the positive control group.

Rutin was dissolved in water to final 10 and 40 mg/mL, Rutin glycoside was dissolved in water to final 13.9 and 55.6 mg/mL and the positive control for platelet aggregation, aspirin was mixed with 1% sodium carboxymethyl cellulose (CMC) to final 5 mg/mL. Positive control for blood coagulation, heparin was mixed with 1% CMC to final 250 IU/mL solution. The test substance was administered once/day for a total of 21 times for 21 days in the stomach using a disposable syringe attached with a sonde for oral administration.

#### 2.7.3. Preparation of Platelet Rich Plasma (PRP) and Platelet Poor Plasma (PPP)

A 7.2 mL blood aliquot was collected from the abdominal vein of the rat and treated with 800 μL of anticoagulant (3.8% sodium citrate). PRP was separated via centrifugation at 950 rpm for 20 min and PPP was obtained by centrifugation at 3000 rpm for 10 min. The number of platelets in PRP was counted using a hemocytometer (Procyte Dx, IDEXX Laboratories, Inc., Westbrook, ME, USA) and diluted to 3.0 × 10^8^/mL with PPP.

#### 2.7.4. Determination of Platelet Aggregation Rate

Platelet aggregation was measured using Chrono-Log aggregometer (Chrono-Log, Haverford Township, PA, USA) according to the method of Lee et al. [23]. An aliquot of 490 μL of the quantified platelets was placed in a cuvette and 10 μL of excipient (water), prepared test substance or positive control was added. After incubation at 37 °C for 10 min, when the baseline stabilized, 10 μL of adenosine diphosphate (ADP) (a platelet aggregation promoter) was added and reacted for 10 min to measure the platelet aggregation rate.

#### 2.7.5. Determination of Blood Coagulation Time

Blood coagulation time was measured using a blood coagulation analyzer (CA660, Sysmex, Kobe, Japan). After 10 μL of excipient aliquot, prepared test substance or positive control was added to 490 μL of PPP, prothrombin time (PT) and activated partial thromboplastin time (APTT) were measured.

### 2.8. Statistical Analysis

Most of the experiments were conducted in triplicate and repeated three times. The values were expressed as mean ± standard deviation, and one-way analysis of variance (ANOVA) was carried out using SPSS software (version 22, SPSS Inc., Chicago, IL, USA). Duncan’s multiple range test was used to test for significant differences between the treatments at *p* < 0.05.

## 3. Results

### 3.1. Preparation of Rutin Glycoside

#### 3.1.1. Enzymatic Conversion of Rutin to Rutin Glycoside

**Figure 2:**
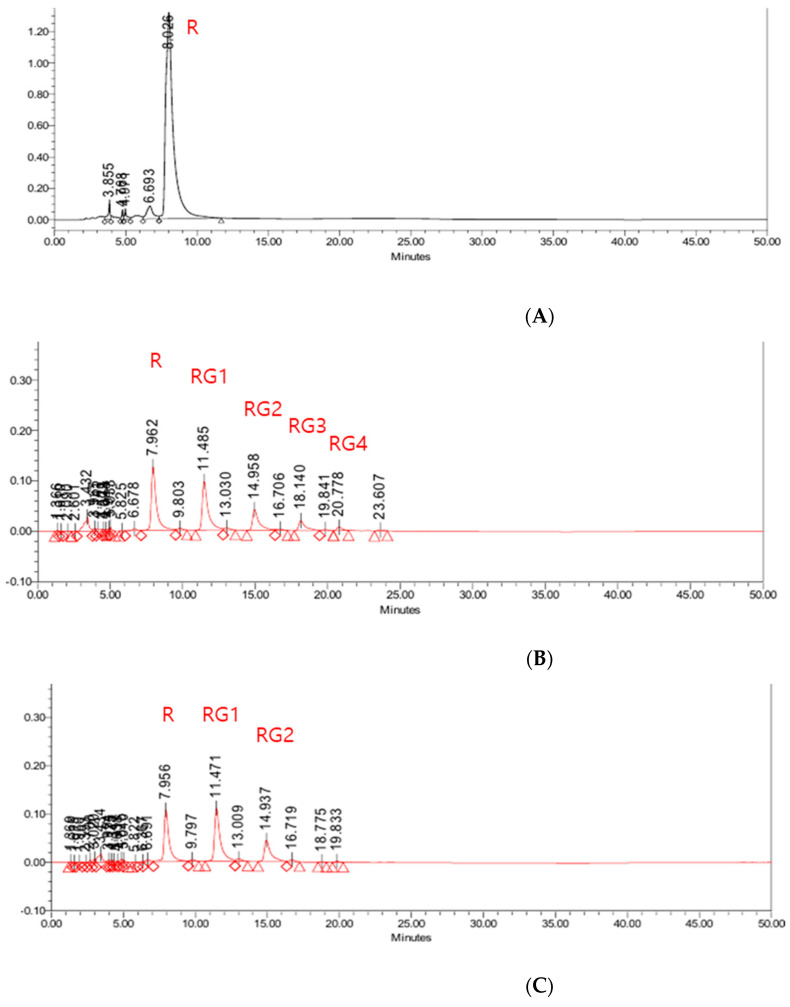
Rutin and rutin glycosides analyzed by HPLC during enzyme reaction. (**A**) Rutin before enzyme reaction, (**B**) rutin poly-glucosides after cyclodextrin glycosyl transferase (CGTase) reaction, (**C**) rutin mono-glucoside and rutin di-glucoside after β-amylase reaction. R; rutin, RG1; rutin mono-glucoside, RG2; rutin di-glucoside, RG3; rutin tri-glucoside, RG4; rutin tetra-glucoside.

The reaction for the synthesis of rutin glycoside was conducted using dextrin as a glucose donor and rutin as an acceptor. Rutin poly-glucoside was obtained after transglycosylation by CGTase as shown in Figure 2. The glucosyl unit bound to rutin was up to 5-6 glucose. The conversion rate of rutin glycoside obtained in this study was 73.21 ± 0.28%. The rutin poly-glucoside was then treated with β-amylase to remove the glucose unit from rutin poly-glucoside, leaving rutin mono-glucoside and rutin di-glucoside.

Rutin mono-glucoside and rutin di-glucoside obtained through a series of enzymatic reactions were separated and purified from unreacted rutin and sugars using a column filled with macroporous adsorption resin. Highly purified rutin glycoside more than 95% content was obtained by the column chromatographic separation and subsequent crystallization in water/ethanol solution.

#### 3.1.2. Mass Spectral Fragmentation of Rutin Glycosides

The rutin glycosides obtained by the enzyme reaction, particularly rutin mono-glucoside and rutin di-glucoside, were identified via LC-MS-MS analysis.

**Figure 3:**
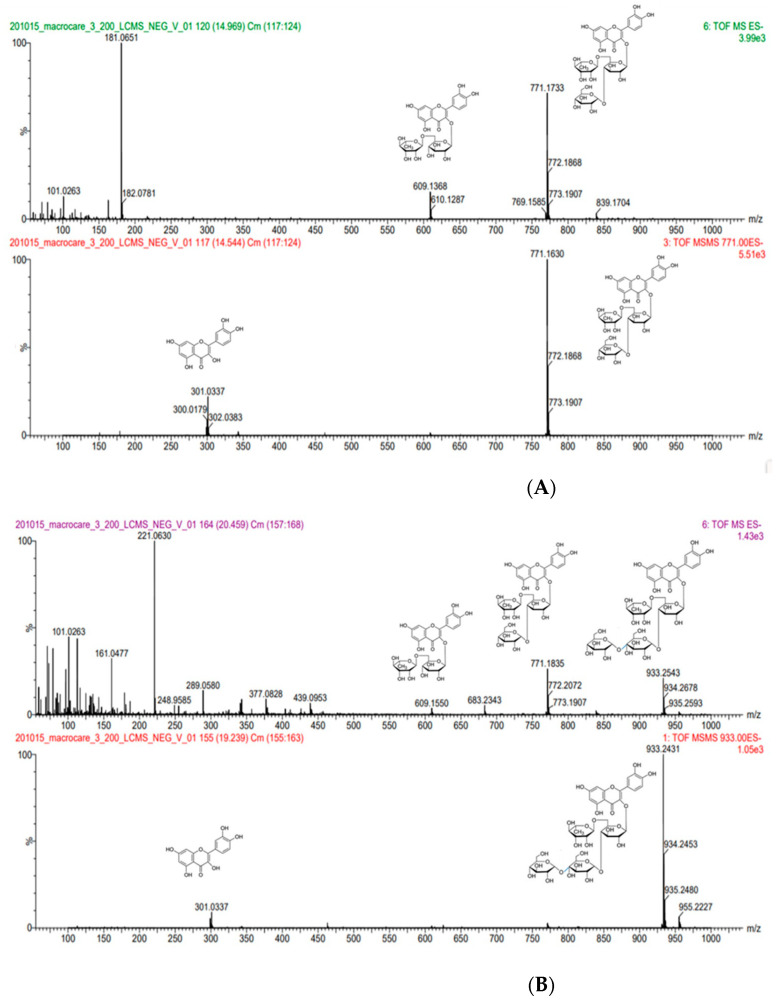
Spectra and fragmentation patterns of rutin mono-glucoside and rutin di-glucoside via LC-MS-MS analysis. (**A**) Mass spectra of rutin mono-glucoside, (**B**) mass spectra of rutin di-glucoside.

The protonated molecular ion of rutin monoglucoside at m/z 771.1733 produced fragment ions at m/z 609.1368 by losing a glucose moiety. The protonated molecular ion of rutin monoglucoside at m/z 771.1630 produced fragment ions at m/z 301.0337 by losing a glucose and rutinose moiety (Figure 3A).

The protonated molecular ion of rutin diglucoside at m/z 933.2543 produced fragment ions at m/z 771.1835 by losing a glucose moiety and 609.1550 by losing a maltose moiety. The protonated molecular ion of rutin diglucoside at m/z 933.2431 produced fragment ions at m/z 301.0337 by losing a maltose and rutinose moiety (Figure 3B).

### 3.2. Partition Coefficient of Rutin and Rutin Glycosides

As described, when the rutin poly-glucoside was treated with glucoamylase, the glucose units were hydrolyzed to leave rutin mono-glucoside. And when the rutin poly-glucoside was treated with β-amylase, the maltose units were hydrolyzed to leave a mixture of rutin mono-glucoside and di-glucoside. For solubility studies, the two rutin glycoside samples were prepared from rutin by transglycosylation and subsequent hydrolysis with different enzymes. The solubility of glycosylated rutin, whether rutin mono-glucoside or rutin mono/di-glucoside, increased more than 10,000 times compared to rutin. The solubility was also confirmed by the octanol–water partition coefficient expressed in Log P. The Log P values of rutin were much higher than those of rutin glycosides, whether rutin mono-glucoside or rutin of mono/di-glucoside mixture (Table 1). The Log P value of the mixture of rutin mono-/di-glucoside was slightly lower than that of rutin mono-glucoside, but was not otherwise significantly different. However, the mixture of rutin mono-/di-glucoside obtained by β-amylase treatment was used as the rutin glycoside for the further experiments in this study.

### 3.3. Antioxidant Activity of Rutin and Rutin Glycoside

**Figure 4:**
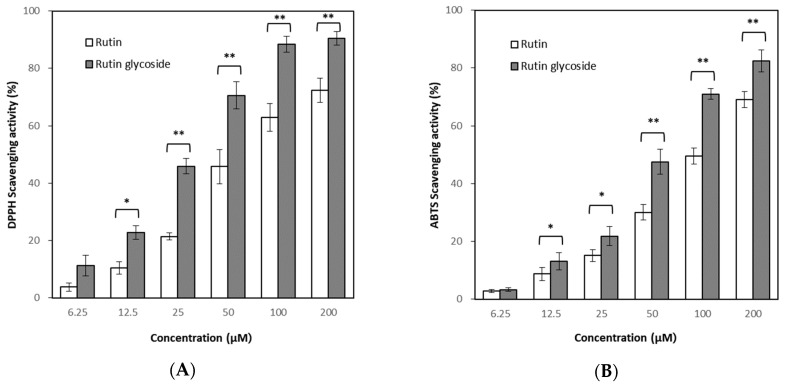
Antioxidant activity of rutin and rutin glycoside. Result expressed as a mean ± SD (*n* = 3). (**A**) 1,1-diphenyl-2-picrylhydrazyl (DPPH) scavenging assay, (**B**) 2,2′-azino-bis-3-ethylbenzthiazoline-6-sulphonic acid (ABTS) scavenging assay. Significant differences were found between the rutin and rutin glycoside (* *p* < 0.05, ** *p* < 0.01).

Antioxidant activity was evaluated by measuring DPPH and ABTS radical scavenging activity, respectively. Treatment of rutin and rutin glycoside showed increased DPPH radical scavenging activity in a concentration-dependent manner (Figure 4A), and rutin glycoside showed higher effects than rutin. The median scavenging concentration (SC_50_) representing 50% of scavenging was identified as 29.13 ± 0.15 μM for rutin glycoside and 60.25 ± 0.09 μM for rutin, respectively, whereas the SC_50_ of ascorbic acid as a positive control was 0.60 ± 0.02 μM.

The results of the ABTS assay were similar to those of the DPPH assay, and the antioxidant effect of rutin glycoside was slightly higher than that of rutin (Figure 4B). The SC_50_ of ABTS assay was found to be 63.21 ± 0.09 μM for rutin glycoside and 105.43 ± 0.16 μM for rutin.

### 3.4. Effects of Rutin and Rutin Glycoside on Cell Viability

**Figure 5:**
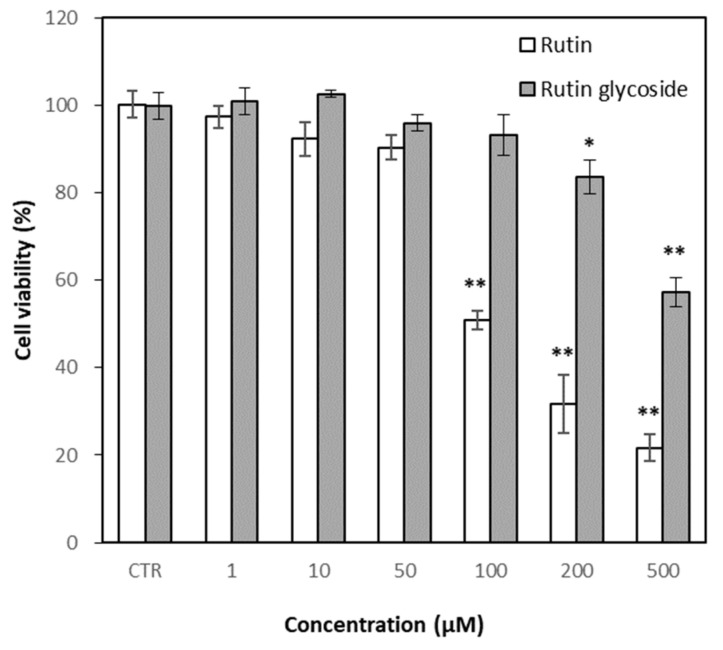
Effect of rutin and rutin glycoside on cell viability. RAW 264.7 cells were treated with rutin and rutin glycoside for 1 h, and incubated with LPS (1 μg/mL) for 24 h. CTR, Control (LPS). Results were represented as mean ± SD (*n* = 3). Significant differences from the control (* *p* < 0.05; ** *p* < 0.01).

The cytotoxic effects of rutin and rutin glycoside were evaluated based on MTT assay before testing their anti-inflammatory effects. Rutin glycoside showed less cytotoxicity than rutin (Figure 5) in RAW 264.7 cells. Rutin glycoside did not induce significant cell death at the concentration above 100 μM, while rutin showed a marked cytotoxic effect at 100 μM. From these results, anti-inflammatory tests were performed up to concentrations of 50 μM for rutin and 100 μM for rutin glycoside.

### 3.5. Effects of Rutin and Rutin Glycoside on NO and PGE2 Levels

**Figure 6:**
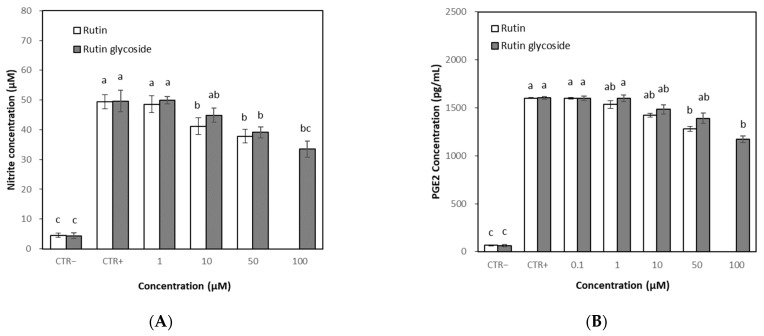

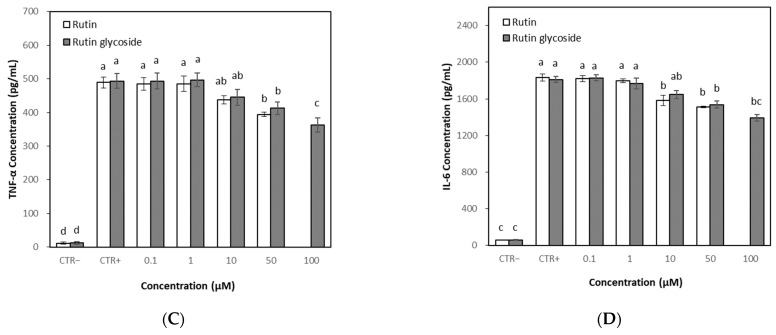
Effect of rutin and rutin glycoside on the production of nitric oxide (NO), Prostaglandin E (PGE) 2, tumor necrosis factor (TNF)-α and interleukin (IL)-6. RAW 264.7 cells were cultured with different rutin and rutin glycoside concentrations for 24 h with LPS (1 μg/mL). CTR−, Negative control. CTR+, Positive control (LPS). Results were represented as mean ± SD (*n* = 3). (**A**) Effect on NO; (**B**) Effect on PGE2; (**C**) Effect on TNF-α; (**D**) Effect on IL-6. Different letters (a, b, c, d) above the bars indicate significant differences (*p* < 0.05), where ab or bc represents the intermediate significance value between a and b or b and c, respectively.

NO and PGE2 are well-known and important inflammatory mediators [24,25,26]. The effects of the rutin and rutin glycoside on NO levels were evaluated in RAW 264.7 cells stimulated with LPS. The treatment of LPS to cells induced significant NO production compared to the untreated control. Cotreatment with rutin or rutin glycoside suppressed NO production at over 10 μM, but the production was not markedly decreased at the tested concentration up to 50 μM (Figure 6A). Rutin and rutin glycoside also suppressed PGE2 production at over 10 μM, but like the effect on NO, the production was not markedly decreased at the tested concentration up to 50 μM (Figure 6B).

### 3.6. Effects of Rutin and Rutin Glycoside on TNF-α and IL-6

The levels of proinflammatory cytokines (such as TNF-α and IL-6) were quantified in the culture medium of RAW 264.7 cells using ELISA to identify the anti-inflammatory properties of rutin and rutin glycoside. The production of TNF-α and IL-6 was markedly increased by LPS stimulation. Co-treatment with rutin or rutin glycoside reduced the cytokines production to some extent, but drastic effects were not observed at tested concentrations up to 50 μg/mL (Figure 6C,D).

### 3.7. Platelet Aggregation and Blood Coagulation In Vitro and In Vivo

#### 3.7.1. Effects of Rutin and Rutin Glycoside on Platelet Aggregation In Vitro

**Figure 7:**
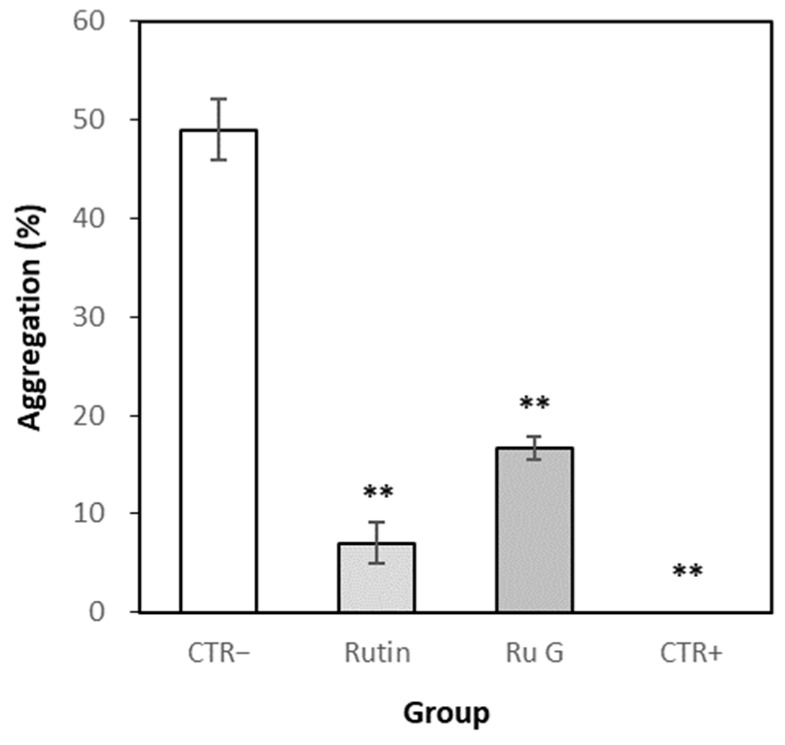
The aggregation rates of platelet-rich plasma (PRP) in vitro. Each time point represented the mean ± S.E. (*n* = 3). CTR−; normal control (distilled water), CTR+; positive control (aspirin), Ru G; rutin glycoside. Significant differences from the normal control group were identified using Independent *t*-test (** *p* < 0.01).

The platelet aggregation rate of the normal control (distilled water) was measured to be 49.0% on average (Figure 7). The platelet aggregation rate of rutin and rutin glycoside averaged 7.0% and 16.7%, respectively, and were statistically significantly decreased compared to the normal control group. The platelet aggregation rate of aspirin, a platelet aggregation inhibitor [27,28] used as a positive control, was 0% on average.

#### 3.7.2. Effects of Rutin and Rutin Glycoside on Blood Coagulation In Vitro

**Figure 8:**
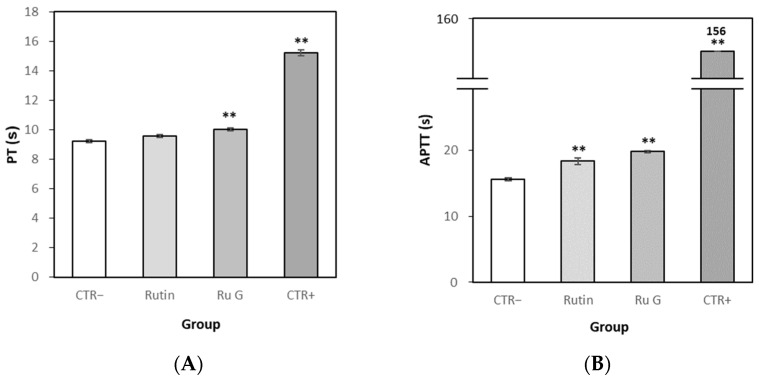
(**A**) Prothrombin time (PT) and (**B**) activated partial thromboplastin time (APTT) of platelet poor plasma (PPP) in vitro. Each time point represented the mean ± S.E. (*n* = 3). CTR− means normal control, CTR+ means positive control (heparin), Ru G means rutin glycoside. Significant differences from the normal control group were determined via Independent *t*-test (** *p* < 0.01).

PT and APTT were investigated as parameters of blood coagulation [29,30]. PT of the normal control, distilled water, was measured to be 9.2 s in the blood coagulation test (Figure 8). The average PT of rutin was 9.6 s and that of rutin glycoside was 10.0 s, which was statistically significantly higher than that of the normal control group. The PT of heparin, a blood coagulation inhibitor used as a positive control, was measured as an average of 15.2 s. In the same condition, APTT of the normal control was measured to be 15.6 s. The average APTT of rutin was 18.4 s, and that of rutin glycoside was 19.8 s. The APTT of heparin was averaged 156.0 s, and aggregation did not occur until the final aggregation measurement time of the instrument. Compared with the normal control group, it was confirmed that PT and APTT of both rutin and rutin glycoside were delayed statistically.

#### 3.7.3. Effects of Rutin and Rutin Glycoside on Platelet Aggregation In Vivo

**Figure 9:**
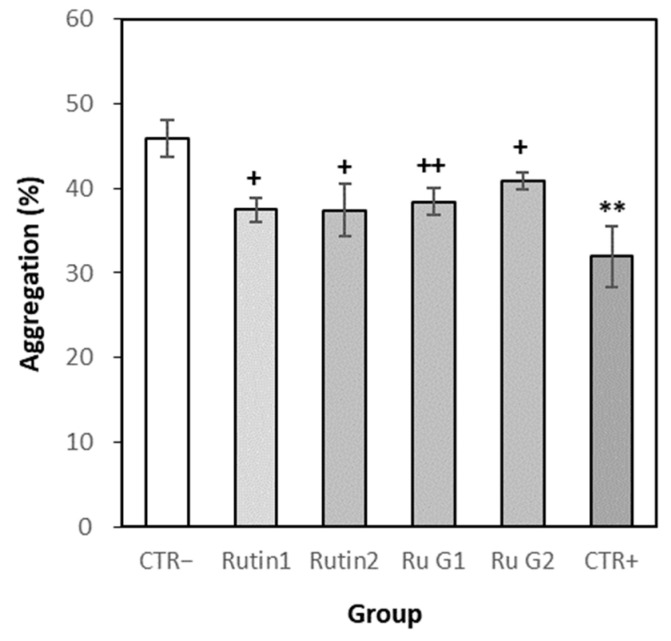
The aggregation rates of PRP in vivo. Each time point represented the mean ± S.E. (*n* = 8). CTR−; normal control, CTR+; positive control (aspirin), Ru G; rutin glycoside. Significant differences from the normal control group were determined via Independent *t*-test (** *p* < 0.01). Significant differences from the normal control group were determined via Dunnett’s *t*-test (+ *p* < 0.05, ++ *p* < 0.01).

The platelet aggregation rate was 45.9% in the normal control group, 32.0% in the positive control group, 37.5% in the group administered Rutin 100 mg/kg, 37.4% in the group Rutin 400 mg/kg, 38.4% in the group Rutin glycoside 139 mg/kg and 40.9% in the group Rutin glycoside 556 mg/kg (Figure 9). The platelet aggregation rate of the rutin and rutin glycoside administration group was significantly decreased compared to the normal control group in vivo.

#### 3.7.4. Effects of Rutin and Rutin Glycoside on Blood Coagulation In Vivo

**Figure 10:**
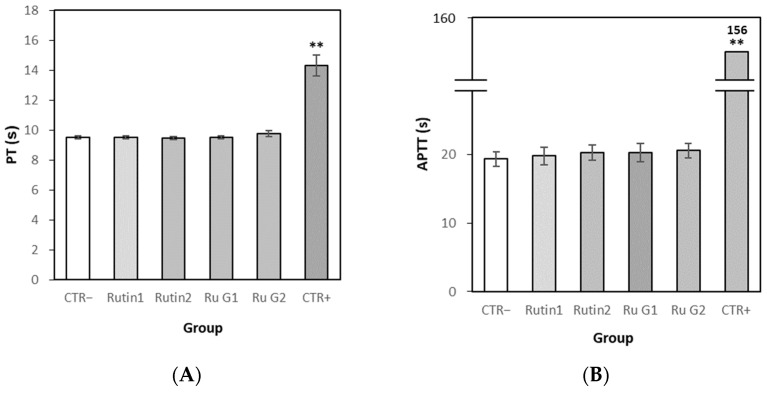
PT (**A**) and APTT (**B**) of PPP in vivo. Each time point represented the mean + S.E. *n* = 8 (G2, *n* = 4). CTR− means normal control, CTR+ means positive control (heparin), Ru G means rutin glycoside. Significant differences from the normal control group were determined via Independent *t*-test (** *p* < 0.01). No statistically significant differences were noted in the substance groups from the normal control group (*p* > 0.05).

In the blood coagulation test, the PT of the normal control group was 9.5 s, that of the positive control group was 14.3 s and those of the test material rutin 100 mg/kg, rutin 400 mg/kg and rutin glycoside 139 mg/kg administration group were all 9.5 s (Figure 10). Contrary to the platelet aggregation rate, there was no significant difference in the rutin or rutin glycoside groups compared to the normal control group. The PT of the rutin glycoside 556 mg/kg administration group was 9.8 s, slightly delayed compared to the normal control group, but this was not statistically significant. The results of APTT were similar to those of PT. The APTT of the normal control group was 19.3 s, those of the positive control group were 156.0 s and those of the test material rutin 100 mg/kg administration group 19.8 s, rutin 400 mg/kg group 20.3 s, rutin glycoside 139 mg/kg group were 20.3 s. The APTT value of the rutin glycoside 556 mg/kg administration group was slightly delayed compared to that of the normal control group at 20.6 s, but it was not statistically significant. In conclusion, the blood coagulation effects of rutin and rutin glycoside were significantly reduced compared to the normal control group in vivo.

## 4. Discussion

Rutin has been found as a component of many plants [31,32]. It is one of the commercially available flavonoids, but the application of rutin has been limited (especially in food and beverage) due to its extremely low water solubility. Moreover, it has been reported that the solubility change of flavonoids could affect biological efficacy [33]. The glycosylation of flavonoids may be a sufficiently efficient and practical solution for the purpose. Rutin glycoside has been developed and used to increase solubility and efficacy; however, there are not many reports comparing various effects of rutin and rutin glycoside at the same time. In this study, we investigated the antioxidant, anti-inflammatory and antithrombotic effects in vitro or in vivo to evaluate the differences between rutin and rutin glycoside with increased solubility.

Flavonoids are known as potent antioxidants, but it has also been reported that flavonoids can act as antioxidants or pro-oxidants depending on their concentration and reaction conditions [34,35]. Therefore, the antioxidant assays, which are related to anti-inflammatory and antithrombotic activity [36], were performed prior to the determination of anti-inflammatory and platelet aggregation activity. In this study, rutin glycoside showed higher activity than rutin in both the DPPH and ABTS radical scavenging assay. The results were similar to those of Yamada et al. [37], who studied the increased antioxidant activity of monoglucosyl rutin at low concentration. The structure and activity relationships of flavonoids as antioxidants were extensively studied [38]; the antioxidant activity of flavonoids depends on the number and location of the hydroxyl moieties, the presence of 2,3-double bond in ring C, 3- and 5-hydroxy groups and the glycosylation model (*C*-glycosides or *O*-glycosides) and position, etc. However, antioxidant activities of flavonoid aglycone and glycoside, and transglycosylated products were variable depending on the compound or condition such as in vitro and in vivo. It was found that 3′ OH group of flavonoids including rutin is essential for antioxidant activity and glycosylation might not affect to the 3′ OH activity [39]. Usually quercetin, the aglycone of rutin, showed higher activity than the naturally glycosylated form (including rutin [40]). On the contrary, the highly increased solubility of rutin by transglycosylation could affect antioxidant activity of rutin [37]. In this study, it was estimated that the difference in antioxidant activity between rutin and rutin glycoside came from the difference in solubility.

Inflammation is an important biological response to restore tissue or body homeostasis. However, excessive inflammation can cause unnecessary collateral damage, resulting in destructive effects [41]. Flavonoids have been known as natural anti-inflammatory agents, and diverse studies have been conducted to understand the pharmacological mechanisms and efficacies of flavonoids, including rutin [42,43]. Rutin and rutin glycoside derived from rutin showed dose-dependent reduction effects on the level of NO or PGE2, and pro-inflammatory cytokines TNF-α and IL-6. Rutin and rutin glycoside did not show much difference in the anti-inflammatory effects, although they showed differences in antioxidant activity. Rather, rutin showed a tendency to reduce anti-inflammatory effects slightly more than rutin glycoside. The effects could not be completed because of the difference in cell viability of the two compounds, but the relatively low cytotoxicity of rutin glycoside would be beneficial for the application of rutin glycosides.

In our body, blood coagulation and dissolution are controlled and in equilibrium. When the balance is disrupted by various intrinsic or extrinsic causes, blood clots form and blood circulation is disturbed, stopping oxygen and nutrients from reaching tissues and organs. Many in vitro and in vivo studies have shown that blood coagulation is closely related to inflammation. Inflammation can lead to local or systemic thrombosis and, conversely, thrombosis can make inflammation worse [44,45]. TNF-α, a pro-inflammatory cytokine that is increased in most inflammation conditions, has been studied to induce complement 3 and platelet activation, contributing to changes in blood coagulation [46,47,48]. Tests measuring platelet aggregation rate and blood coagulation time are frequently performed in vitro and in vivo to study the hemostatic activity or antithrombotic effect of compounds [49,50]. Rutin and various flavonoids have been studied to effectively suppress platelet activation and thrombosis, despite some differences in bioavailability [51,52,53]. In this study, rutin glycoside showed antioxidant and anti-inflammatory effects similar to or slightly different from rutin, indicating its potential for antithrombotic effects. Subsequently, platelet aggregation and blood coagulation were investigated in vitro and in vivo to compare and estimate the antithrombotic effects of rutin and rutin glycoside. Treatment of rutin and rutin glycoside similarly reduced platelet aggregation rate and delayed PT and APTT in blood coagulation in vitro. The repeated administration of rutin and rutin glycoside for 21 days was also effective on platelet aggregation rate in vivo. However, unlike in vitro, the PT and APTT of both the rutin and rutin glycoside group was not significantly delayed in an in vivo blood coagulation test.

It has been reported that the bioavailability of many flavonoids differ between in vitro and in vivo results. High antioxidant capacity of apple extracts in vitro was compared with failure of inhibition of lipid oxidation in vivo, and the inconsistence is presumed to the poor absorption and metabolic conversion of apple polyphenols [54]. Chrysin showed many health-promoting effects including antioxidant and anti-inflammatory activities in vitro, but its exceedingly low absorption after oral administration limited its therapeutic applications [55]. Regarding the difference in the bioavailability of rutin and rutin glycoside in vitro and in vivo, further studies are needed on experimental conditions, such as prolonged administration time or increased dose and metabolic processes.

## 5. Conclusions

A rutin glycoside consisting of rutin mono-glucoside and di-glucoside was prepared from rutin by enzymatic transglycosylation. The rutin glycoside showed much higher water solubility than rutin and showed a higher effect than rutin in antioxidant assays. However, they were similar in the reduction of inflammatory mediators and pro-inflammatory cytokines in vitro, platelet aggregation rate and blood coagulation both in vitro and in vivo. Comparing in vitro and in vivo effects, rutin and rutin glycoside were similarly effective on the platelet aggregation both in vitro and in vivo, but they were effective only for blood coagulation in vitro (but not in vivo).

## Figures and Tables

**Table 1:** Octanol/water partition coefficients of rutin and rutin glycosides.

Compound	Log P_o/w_
Rutin	0.61 ± 0.02 ^a^
Rutin mono-glucoside Rutin mono-/di-glucoside	−1.40 ± 0.01 ^b^ −1.45 ± 0.02 ^b^

Results were represented as a mean ± SD (*n* = 3). Different letters (a and b) in the column are significant differences according to Duncan’s multiple range test (*p* < 0.05).

## Data Availability

Data is contained within the article.
